# Supplementary material for: An invisible ubiquitin conformation is required for efficient phosphorylation by PINK1
Source: EMBO J. 2017 Nov 13;36(24):3555–72. doi: 10.15252/embj.201797876 (PMC5730886; doi:10.15252/embj.201797876)
Supplement: Supplementary file 1 — Appendix [file EMBJ-36-3555-s001.pdf]

# An invisible ubiquitin conformation is required for efficient phosphorylation by PINK1

# Appendix

Christina Gladkova[$], Alexander F. Schubert[$], Jane L. Wagstaff, Jonathan N. Pruneda, Stefan M.V. Freund and David Komander*

Medical Research Council Laboratory of Molecular Biology, Francis Crick Avenue, Cambridge, CB2 0QH, UK.

* Corresponding author: David Komander, dk@mrc-lmb.cam.ac.uk
$ These authors contributed equally to this work

Running title: Ub-CR is a superior PINK1 substrate

TABLE OF CONTENTS:

Appendix Fig. S1. CEST experiment full spectra
Appendix Fig. S2. BEST TROSY spectrum of Ub L67S
Appendix Fig. S3. BEST TROSY spectra of Ub TVLN and phosphoUb TVLN
Appendix Fig. S4. BEST TROSY spectra of Ub L71Y and phosphoUb L71Y
Appendix Fig. S5. BEST TROSY spectra of Ub F4A and phosphoUb F4A
Appendix Fig. S6. $^{15}N\{^{1}H\}$ hetNOE experiments on Ub variants
Appendix Fig. S7. CLEANEX experiments on Ub variants
Appendix Fig. S8. Representative CEST fits
Appendix Fig. S9. PINK1 interaction analysis
Appendix Fig. S10. PINK1 interaction analysis
Appendix Fig. S11. PINK1 interaction analysis
Appendix Fig. S12. PINK1 interaction analysis in the presence of MgAMP-PNP
Appendix Fig. S13. PINK1 interaction analysis with phosphoUb

# Appendix Figure S1

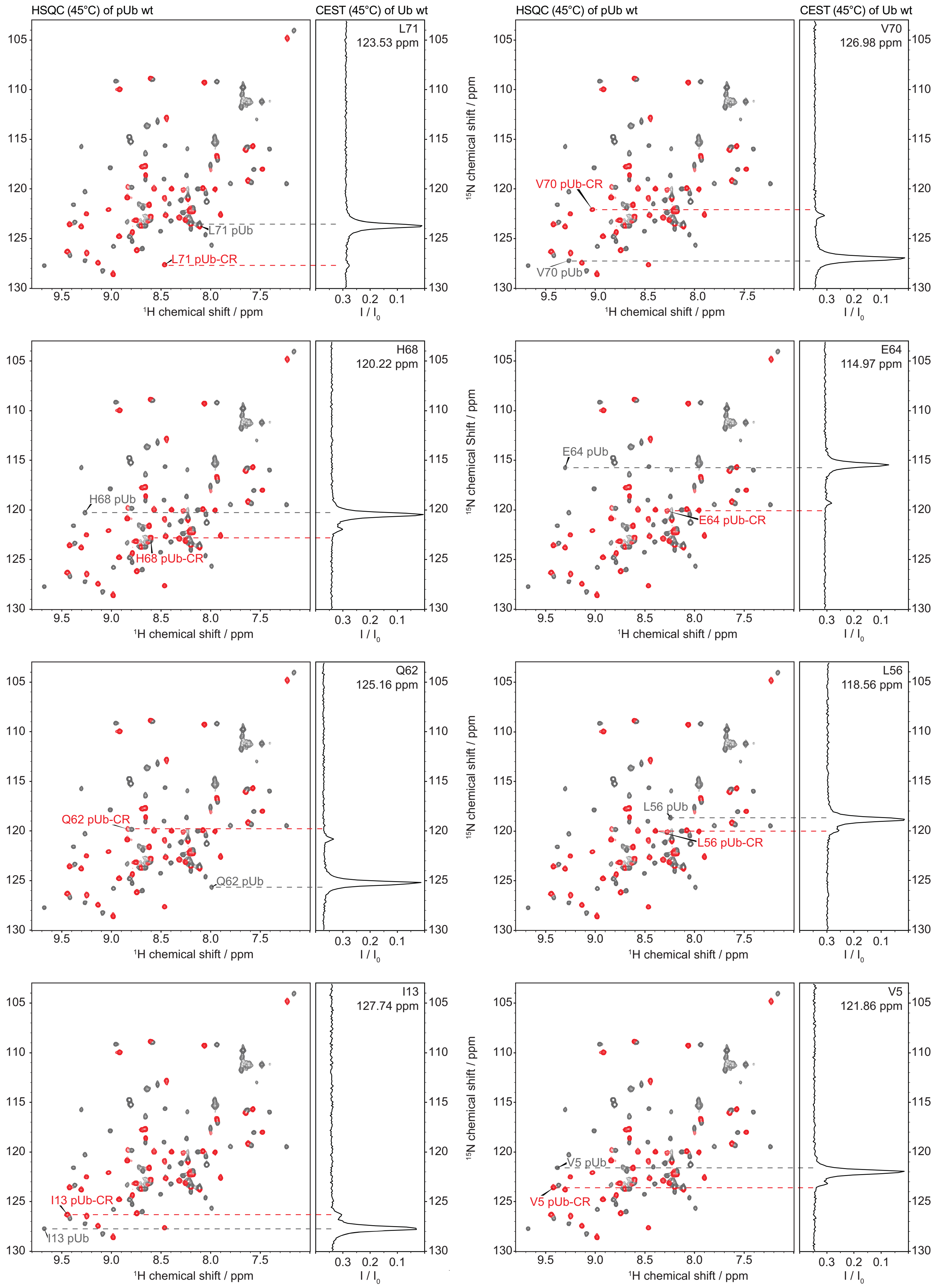

**Appendix Figure S1. CEST experiment full spectra**

Full CEST $^{15}N$ profiles are shown alongside phosphoUb HSQC spectra. Residues targeted by the $^{15}N$ frequency are indicated, and corresponding HSQC positions of the phosphoUb and phosphoUb-CR resonances are indicated.

# Appendix Figure S2

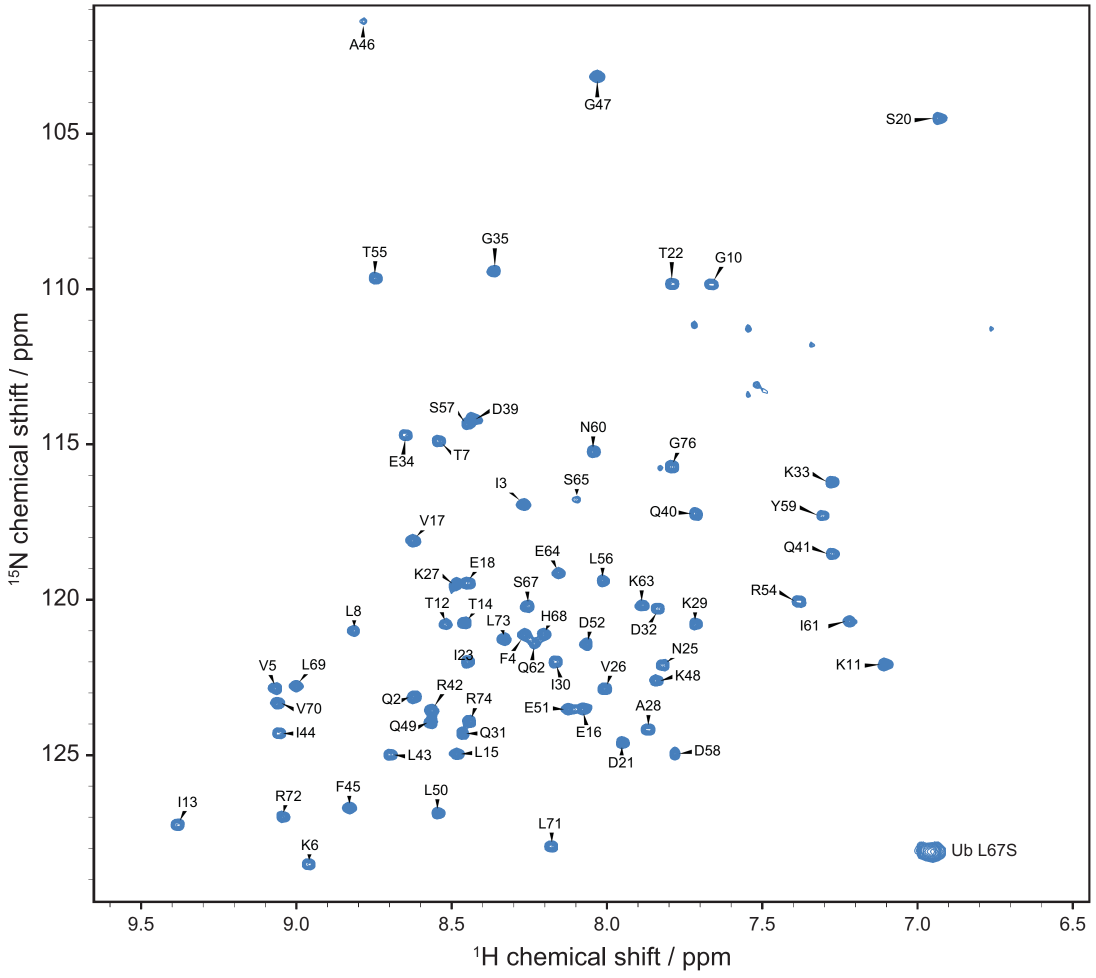

**Appendix Figure S2. BEST TROSY spectrum of Ub L67S**

Complete and reassigned BEST TROSY spectrum of $^{13}C$, $^{15}N$-labelled Ub L67S.

# Appendix Figure S3

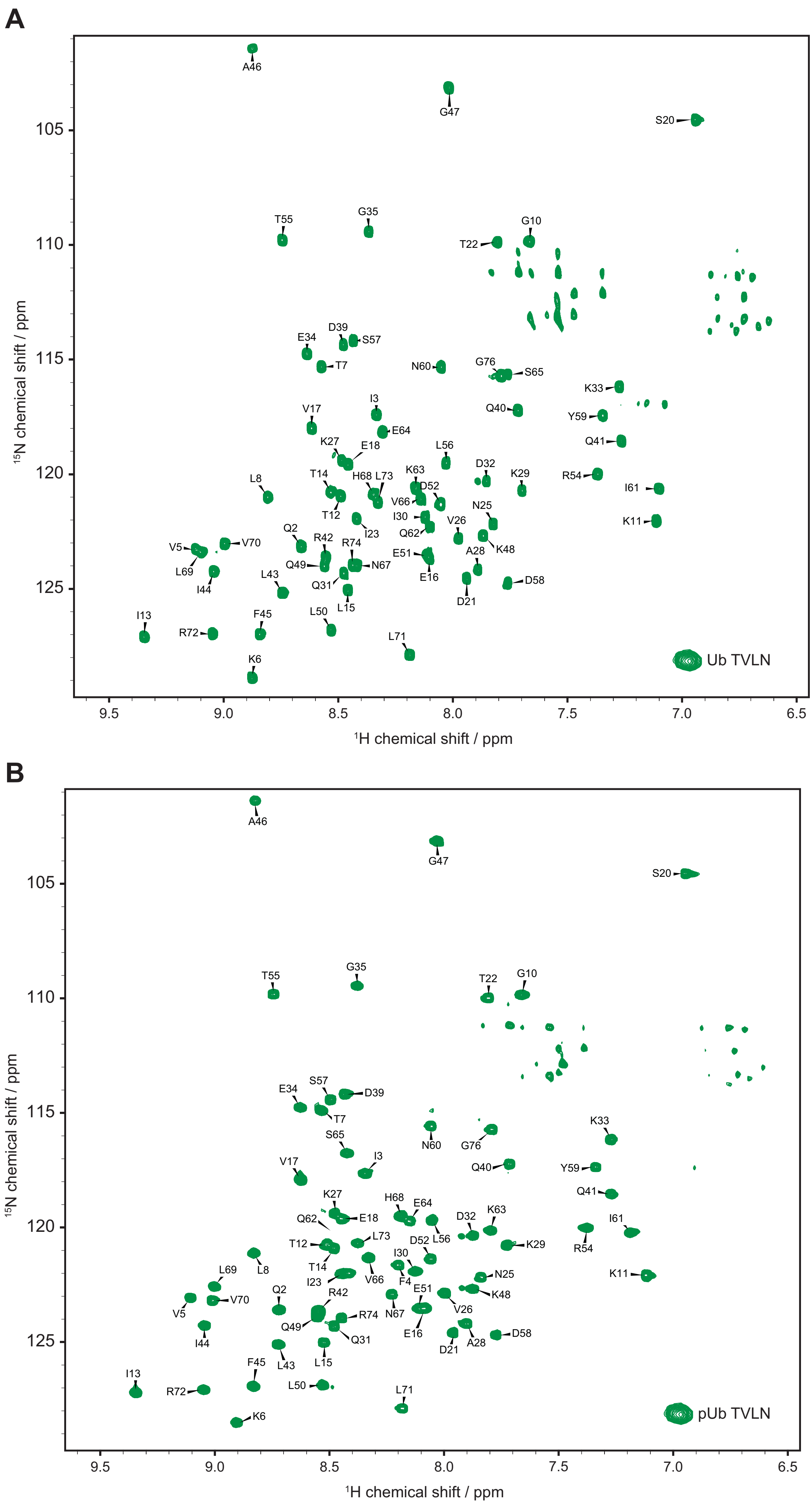

**Appendix Figure S3. BEST TROSY spectra of Ub TVLN and phosphoUb TVLN**

Complete and reassigned BEST TROSY spectra of $^{13}C$, $^{15}N$-labelled Ub TVLN (A) and phosphoUb TVLN (B).

# Appendix Figure S4

**A**

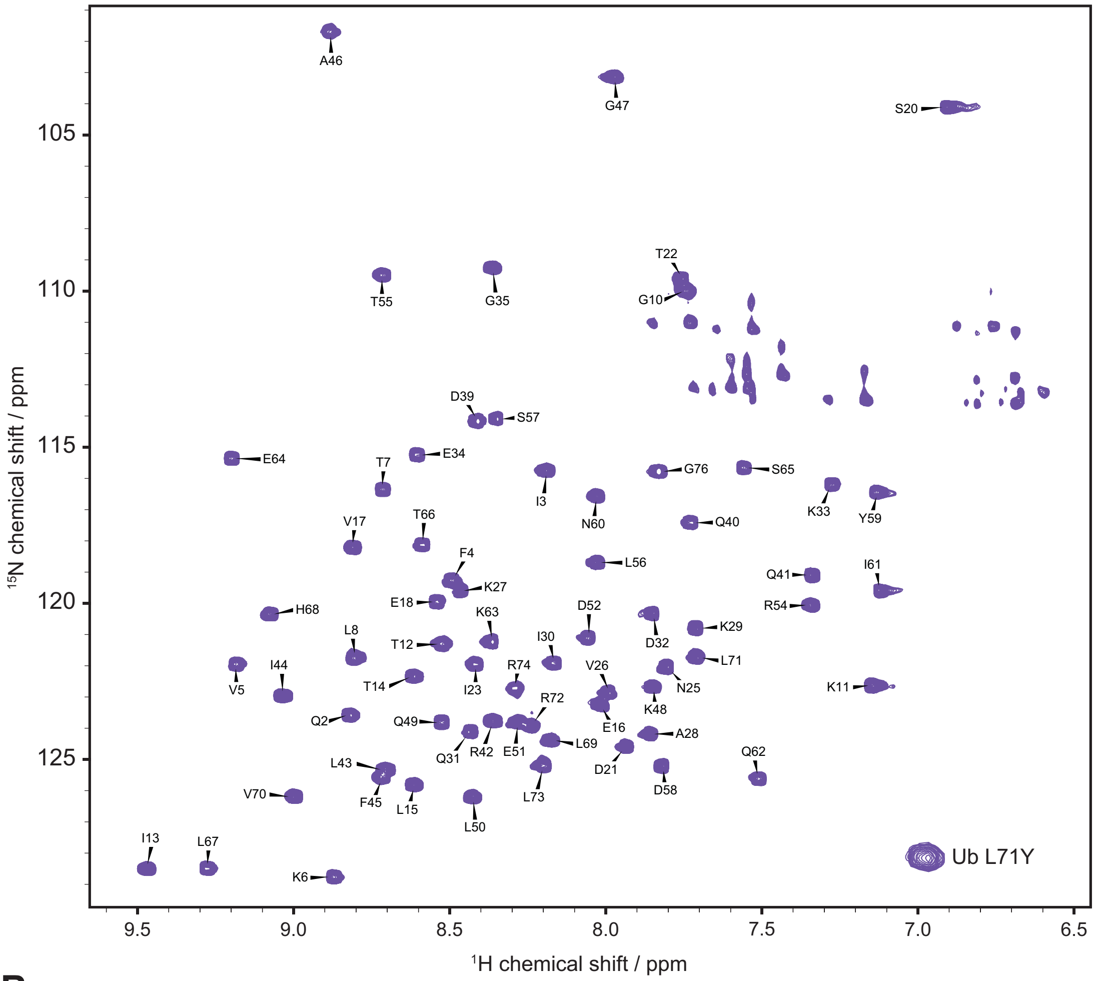


**B**

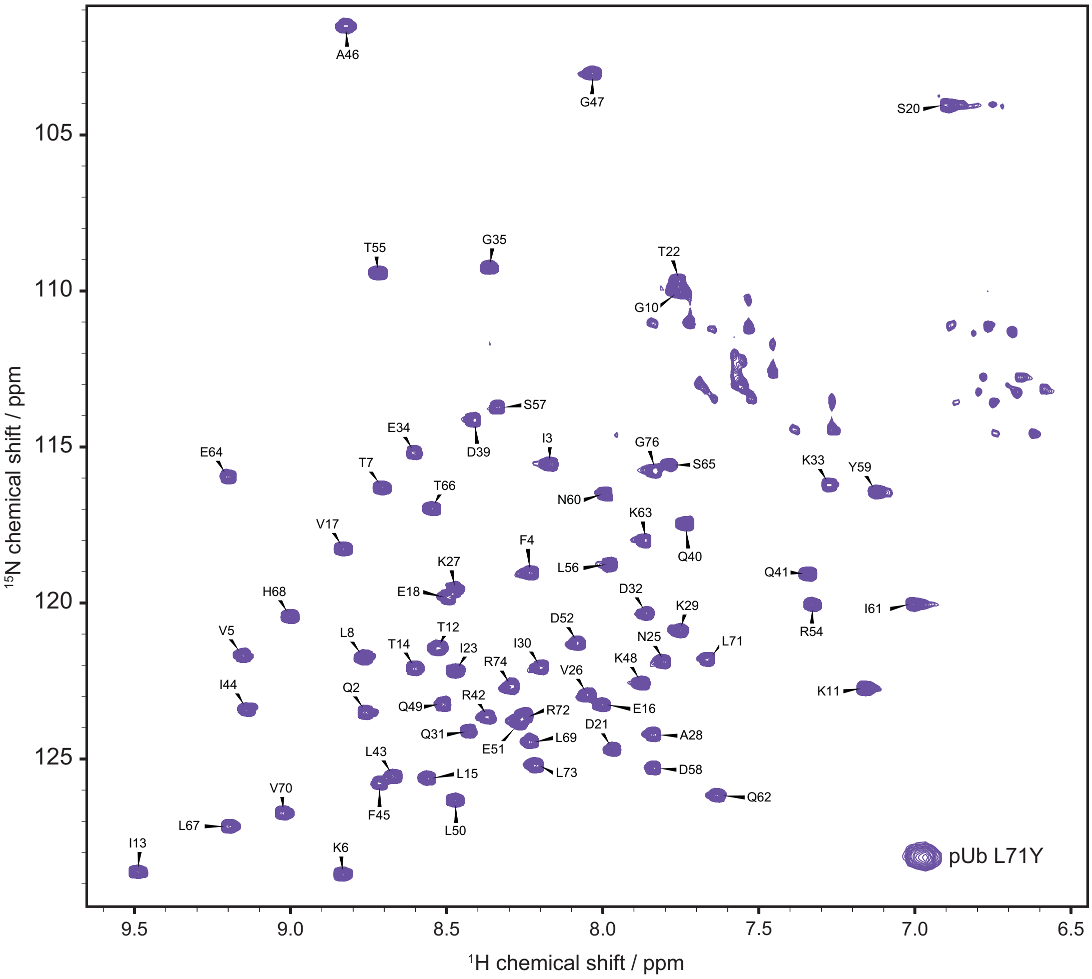

**Appendix Figure S4. BEST TROSY spectra of Ub L71Y and phosphoUb L71Y**

Complete and reassigned BEST TROSY spectra of $^{13}C$, $^{15}N$-labelled Ub L71Y (A) and phosphoUb L71Y (B).

# Appendix Figure S5

A

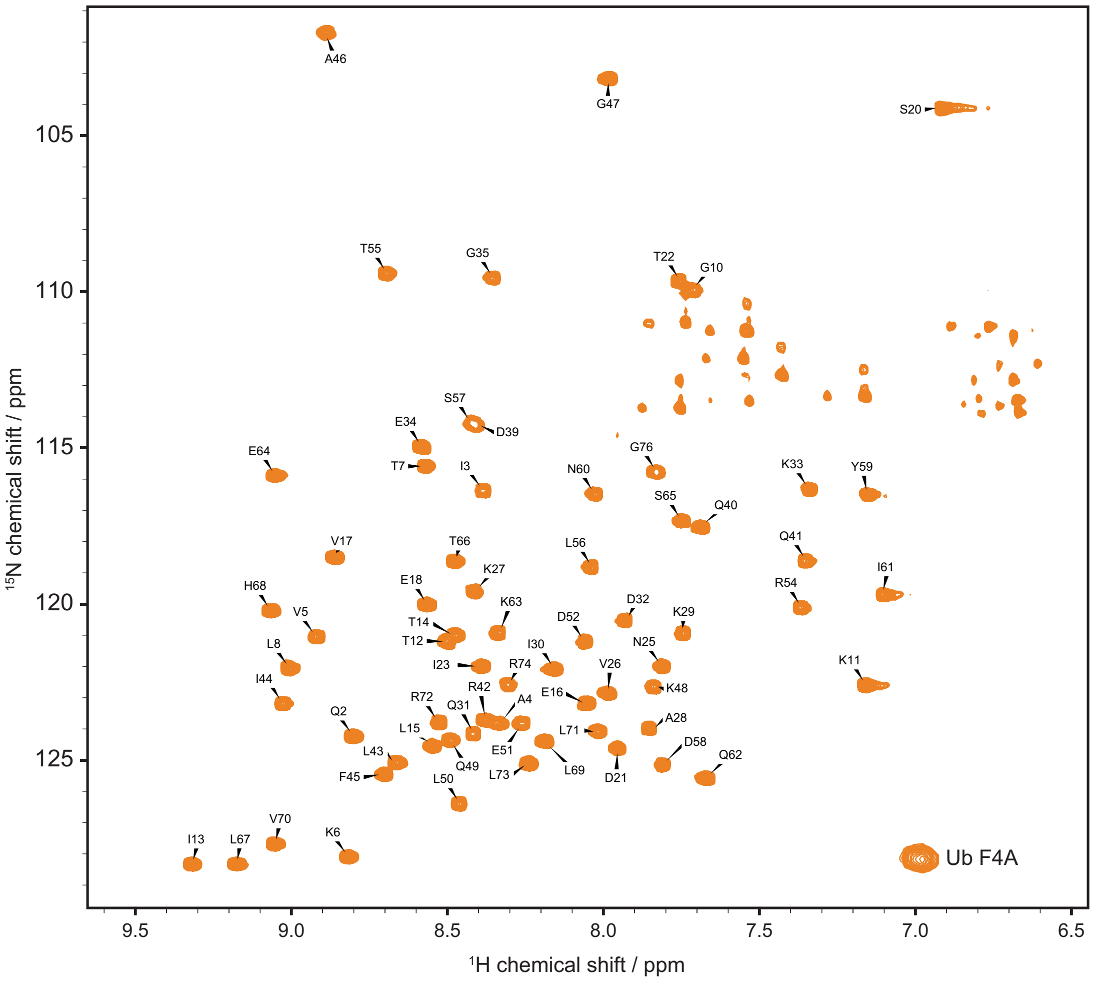


B

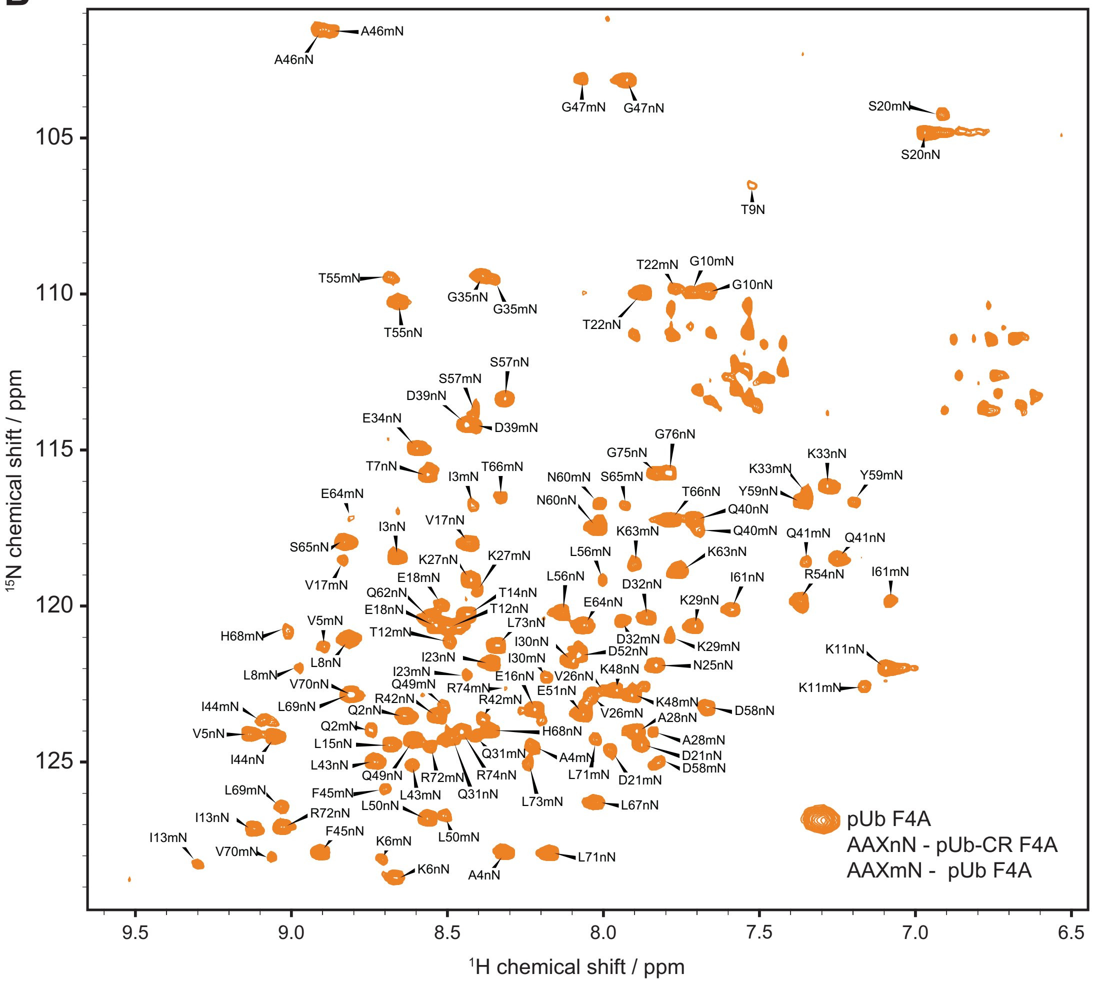

**Appendix Figure S5. BEST TROSY spectra of Ub F4A and phosphoUb F4A**

Complete and reassigned BEST TROSY spectra of $^{13}C$, $^{15}N$-labelled Ub F4A (A) and phosphoUb F4A (B).

# Appendix Figure S6

$^{15}$N{$^{1}$H} NOE (heteronuclear NOE)
(unphosphorylated species)

$^{15}$N{$^{1}$H} NOE (heteronuclear NOE)
(phosphorylated species)

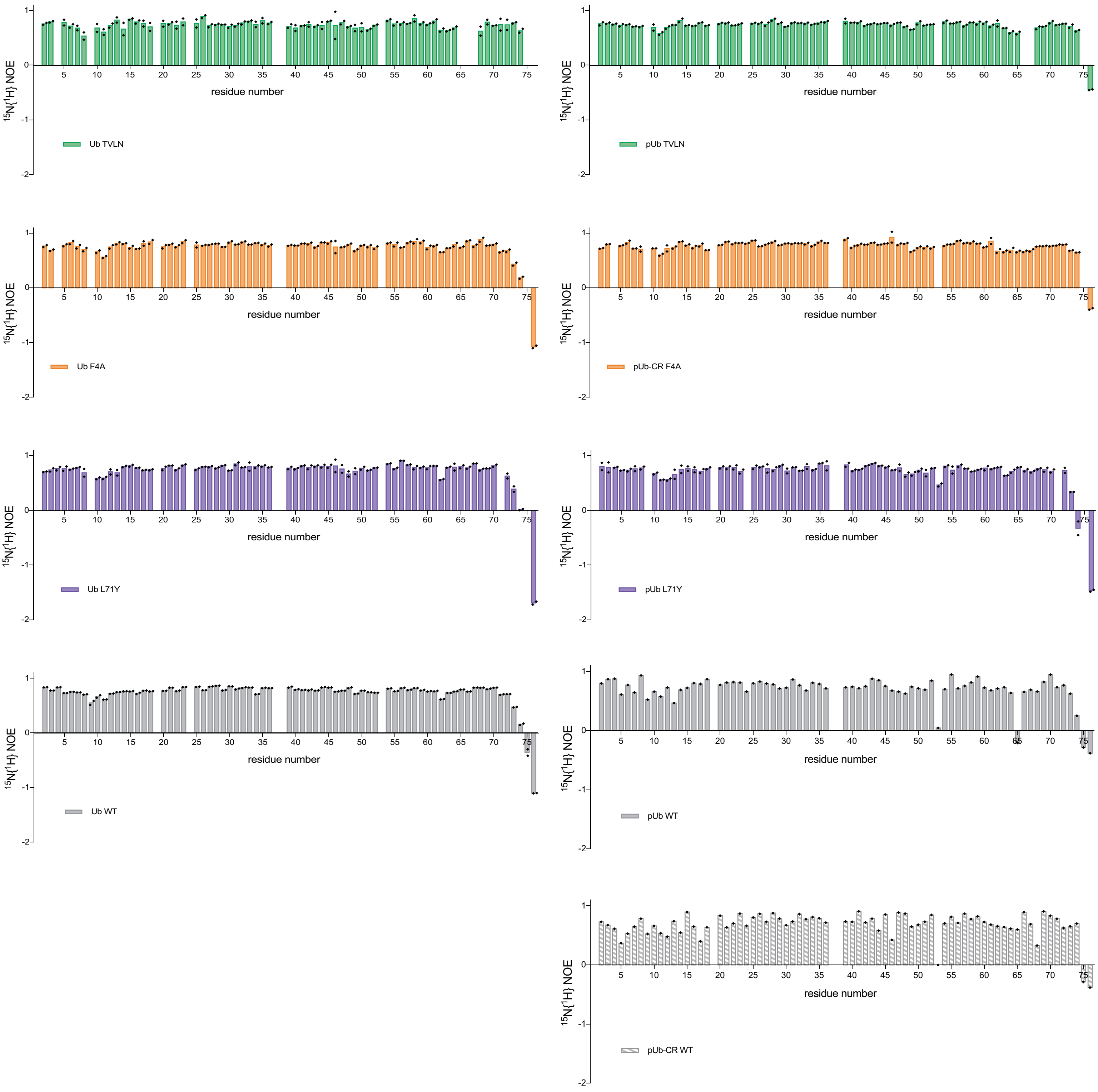

**Appendix Figure S6. $^{15}N\{^1H\}$ hetNOE experiments on Ub variants**

Complete set of $^{15}N\{^1H\}$ hetNOE measurements of Ub residues for indicated species. Data for pUb and pUb-CR WT are replotted from (Wauer *et al*, 2015a).

# Appendix Figure S7

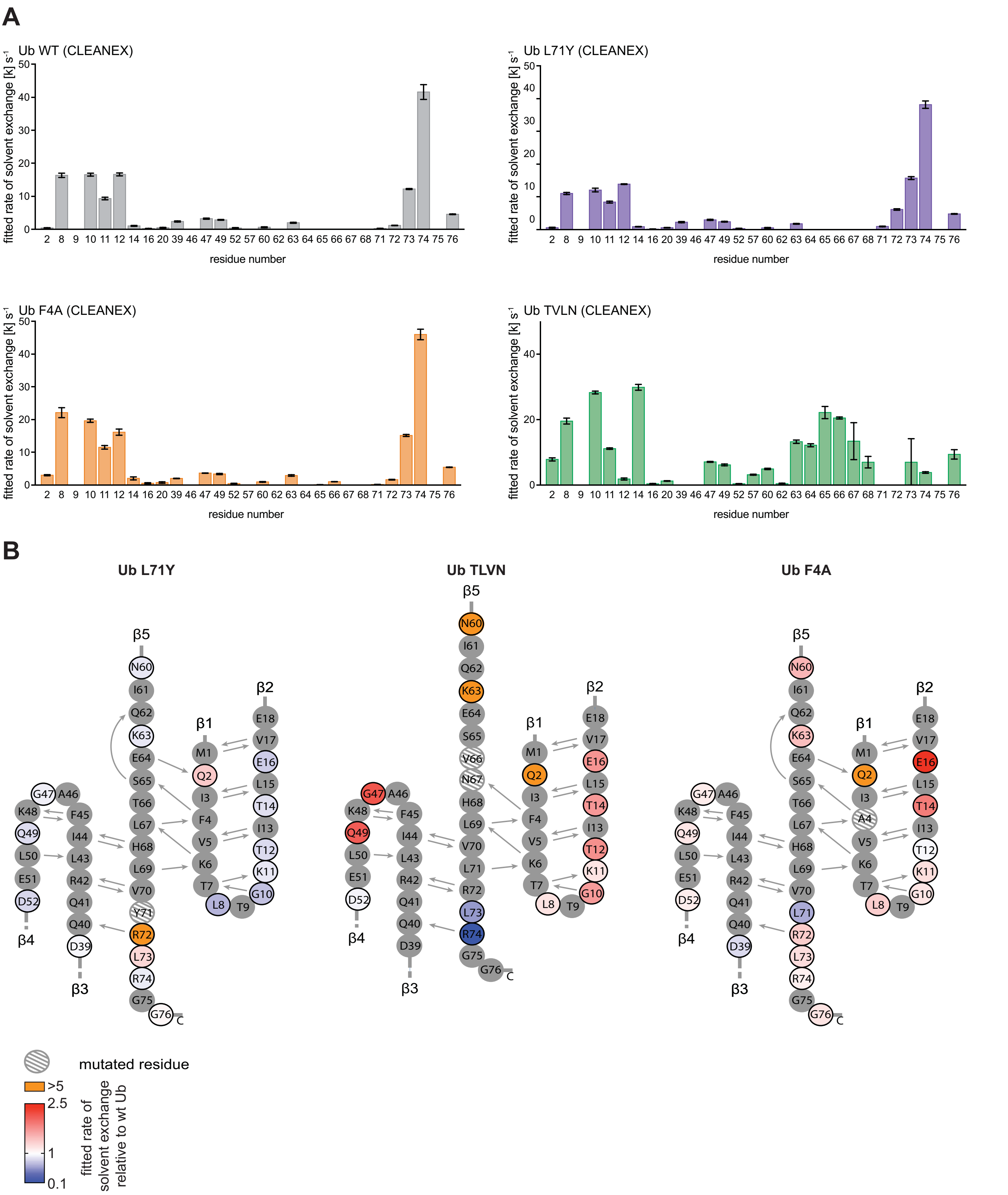

**Appendix Figure S7. CLEANEX experiments on Ub variants**

A) Bar graph showing the fitted rate of solvent exchange for indicated Ub residues.
B) Hydrogen bonding pattern of Ub L71Y (in common Ub conformation), Ub TVLN (in Ub-CR conformation) and Ub F4A (in common Ub conformation) colouring residues with increased or decreased rate of solvent exchange as compared to wt Ub.

# Appendix Figure S8

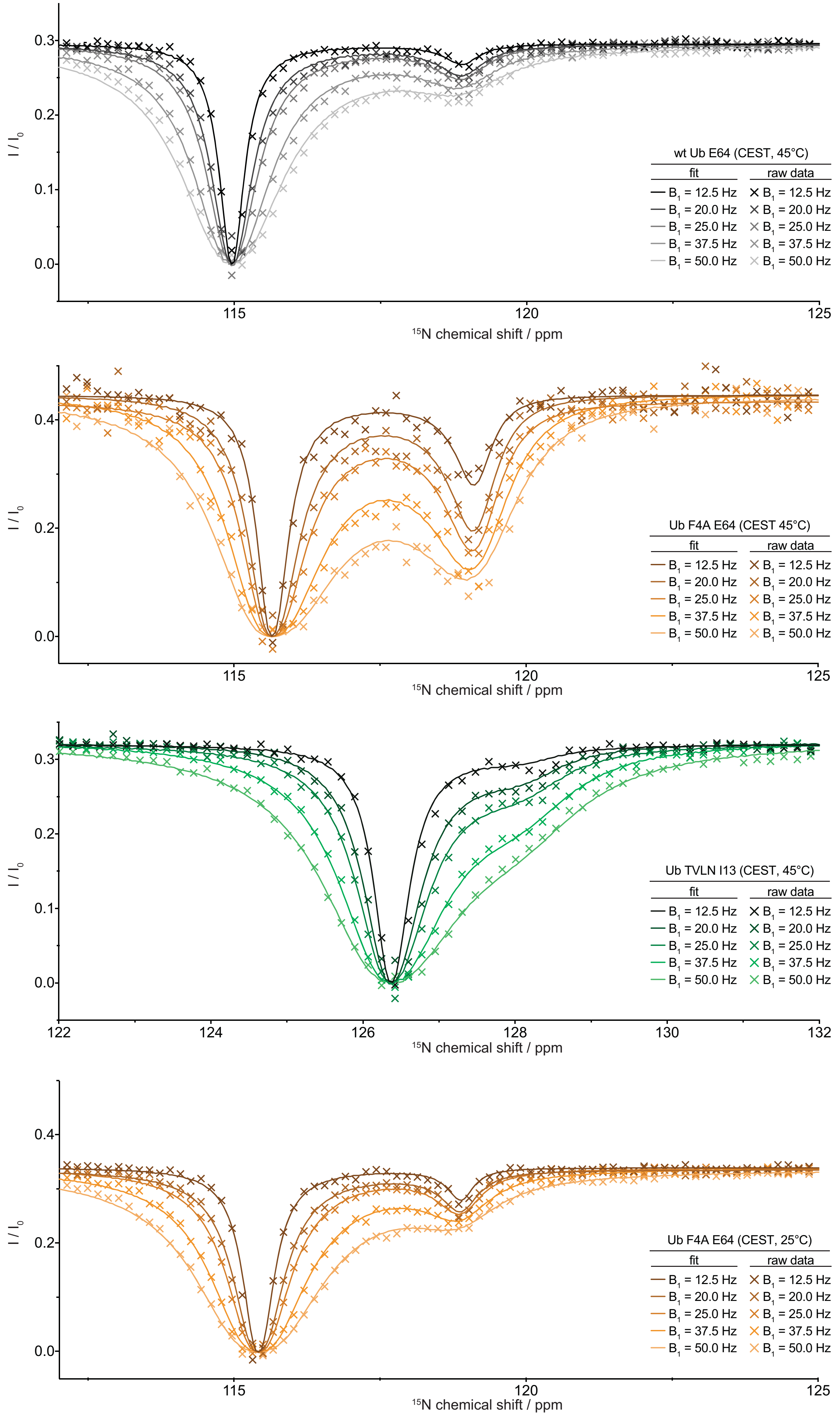

**Appendix Figure S8. Representative CEST fits**

Representative peak fits for CEST experiments on wt Ub, Ub TVLN, and Ub F4A at different $B_1$ fields.

# Appendix Figure S9

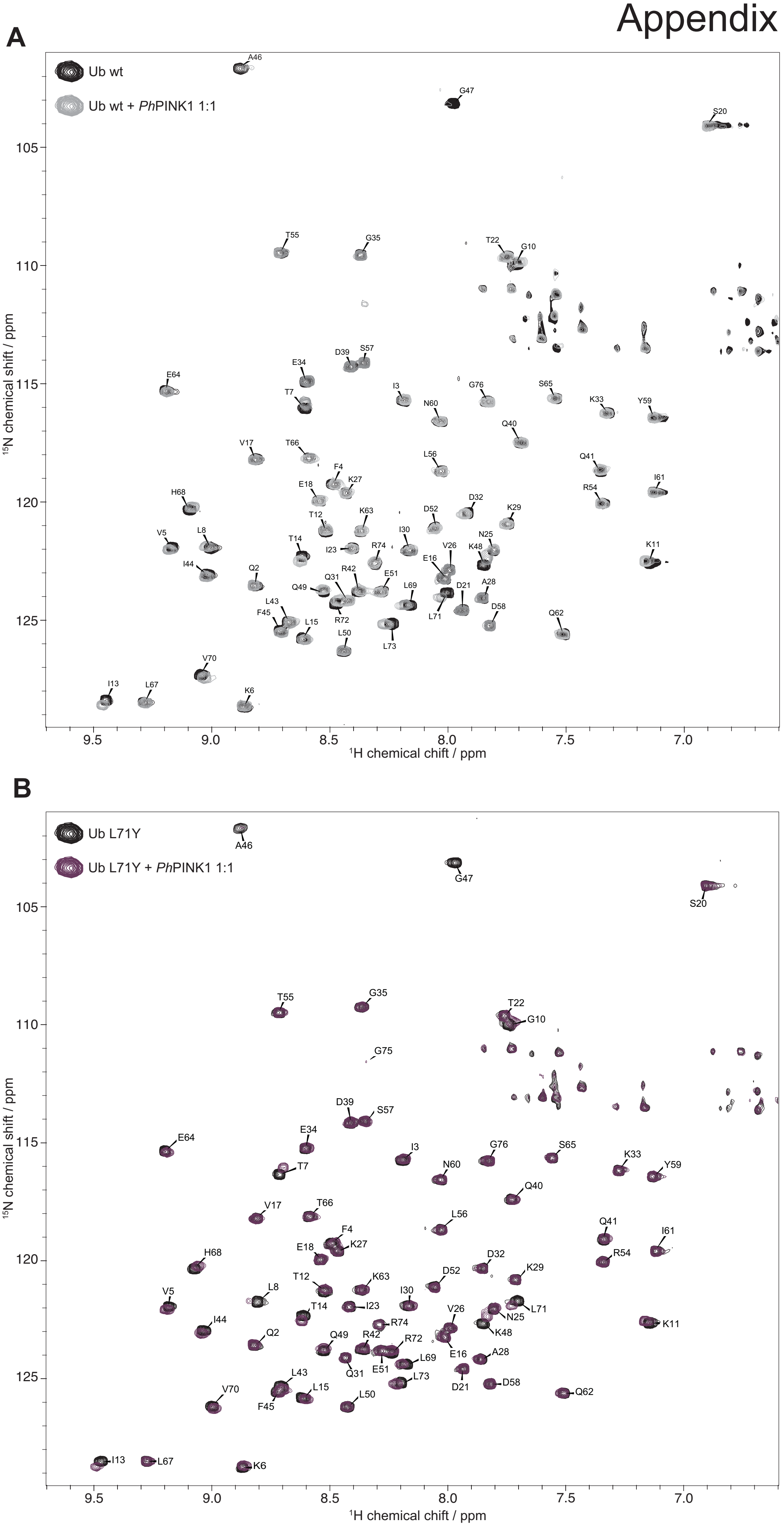

**Appendix Figure S9. PINK1 interaction analysis**

Complete BEST TROSY spectral overlay in the absence and presence of equimolar *Ph*PINK1 for wt Ub (A) and Ub L71Y (B).

# Appendix Figure S10

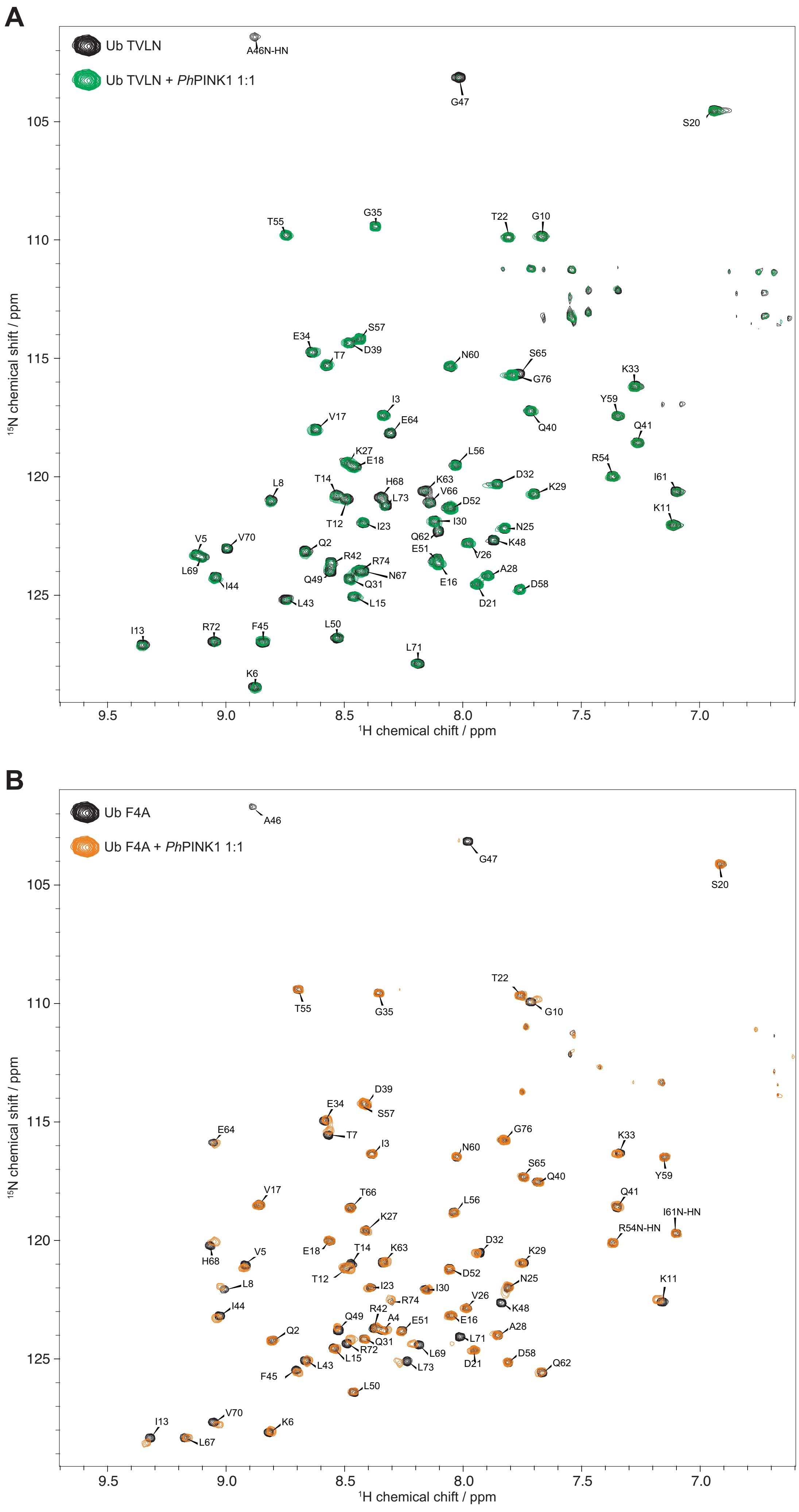

**Appendix Figure S10. PINK1 interaction analysis**

Complete BEST TROSY spectral overlay in the absence and presence of equimolar *Ph*PINK1 for Ub TVLN (A) and Ub F4A (B).

# Appendix Figure S11

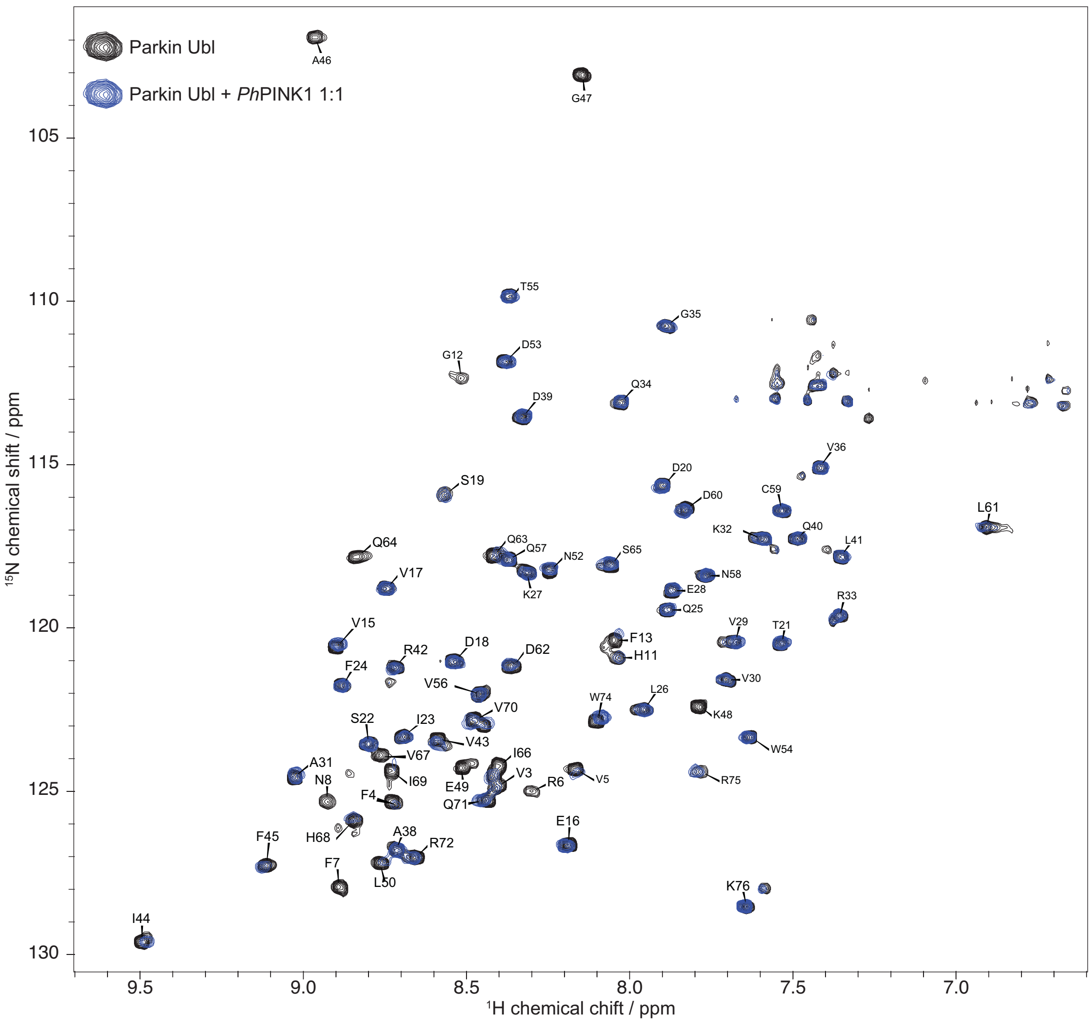

**Appendix Figure S11. PINK1 interaction analysis**

Complete BEST TROSY spectral overlay in the absence and presence of equimolar *Ph*PINK1 for Parkin Ubl.

# Appendix Figure S12

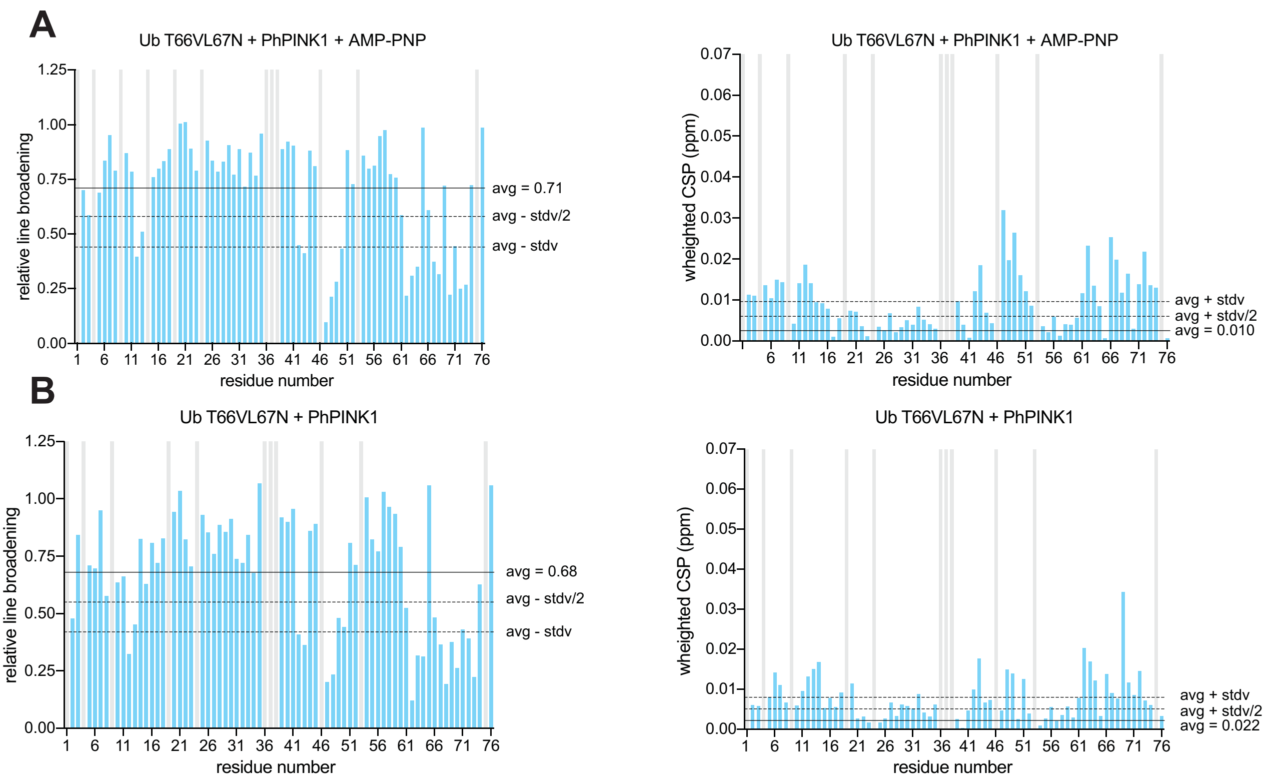

**Appendix Figure S12. PINK1 interaction analysis in the presence of MgAMP-PNP**

Line broadening and chemical shift perturbation analyses of the Ub TVLN interaction with *Ph*PINK1 in the presence (A) and absence (B) of MgAMP-PNP.

# Appendix Figure S13

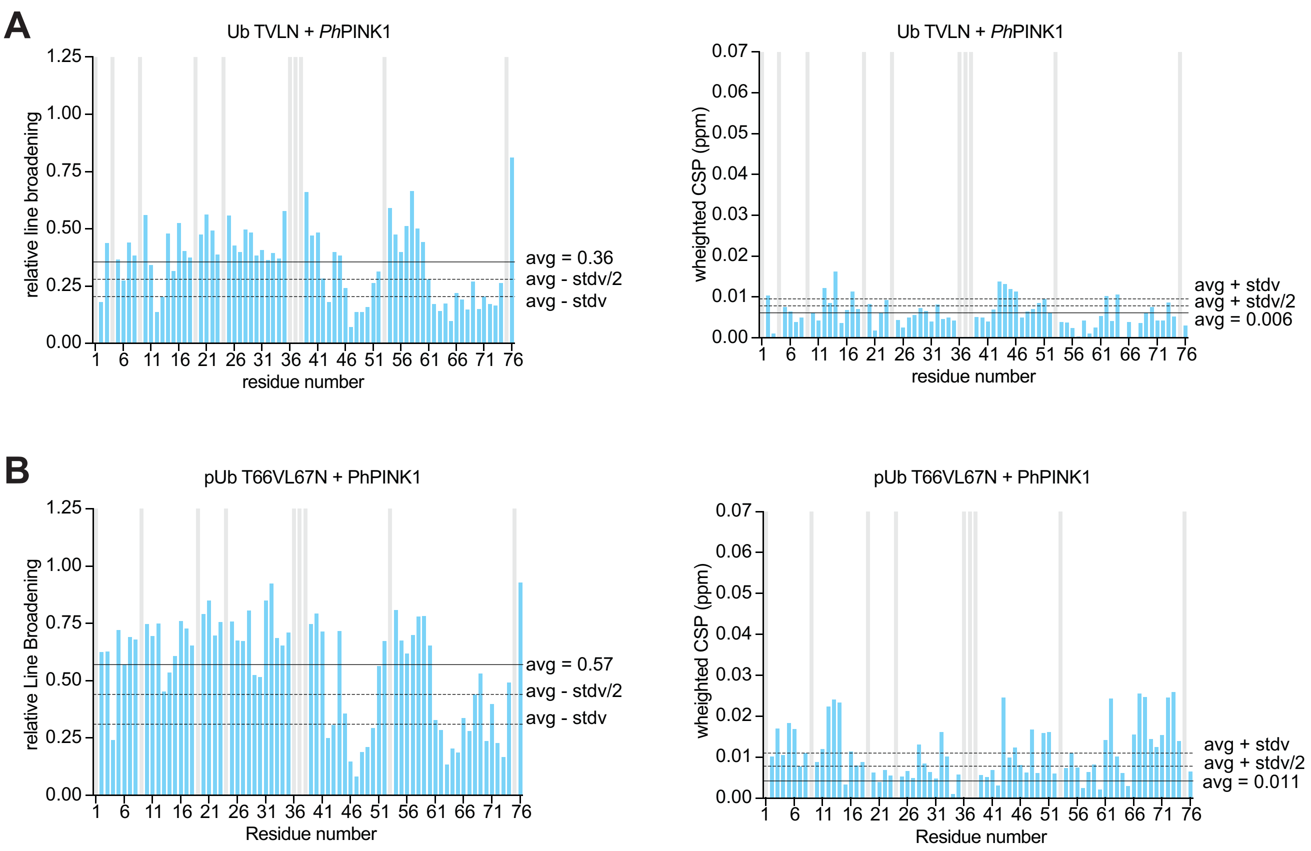

**Appendix Figure S13. PINK1 interaction analysis with phosphoUb**

Line broadening and chemical shift perturbation analyses of the Ub TVLN (A) and phosphoUb TVLN (B) interaction with *Ph*PINK1.
