# Supplementary material for: An invisible ubiquitin conformation is required for efficient phosphorylation by PINK1
Source: EMBO J. 2017 Nov 13;36(24):3555–72. doi: 10.15252/embj.201797876 (PMC5730886; doi:10.15252/embj.201797876)
Supplement: Supplementary file 2 — Expanded View Figures PDF [file EMBJ-36-3555-s002.pdf]

Expanded View Figures

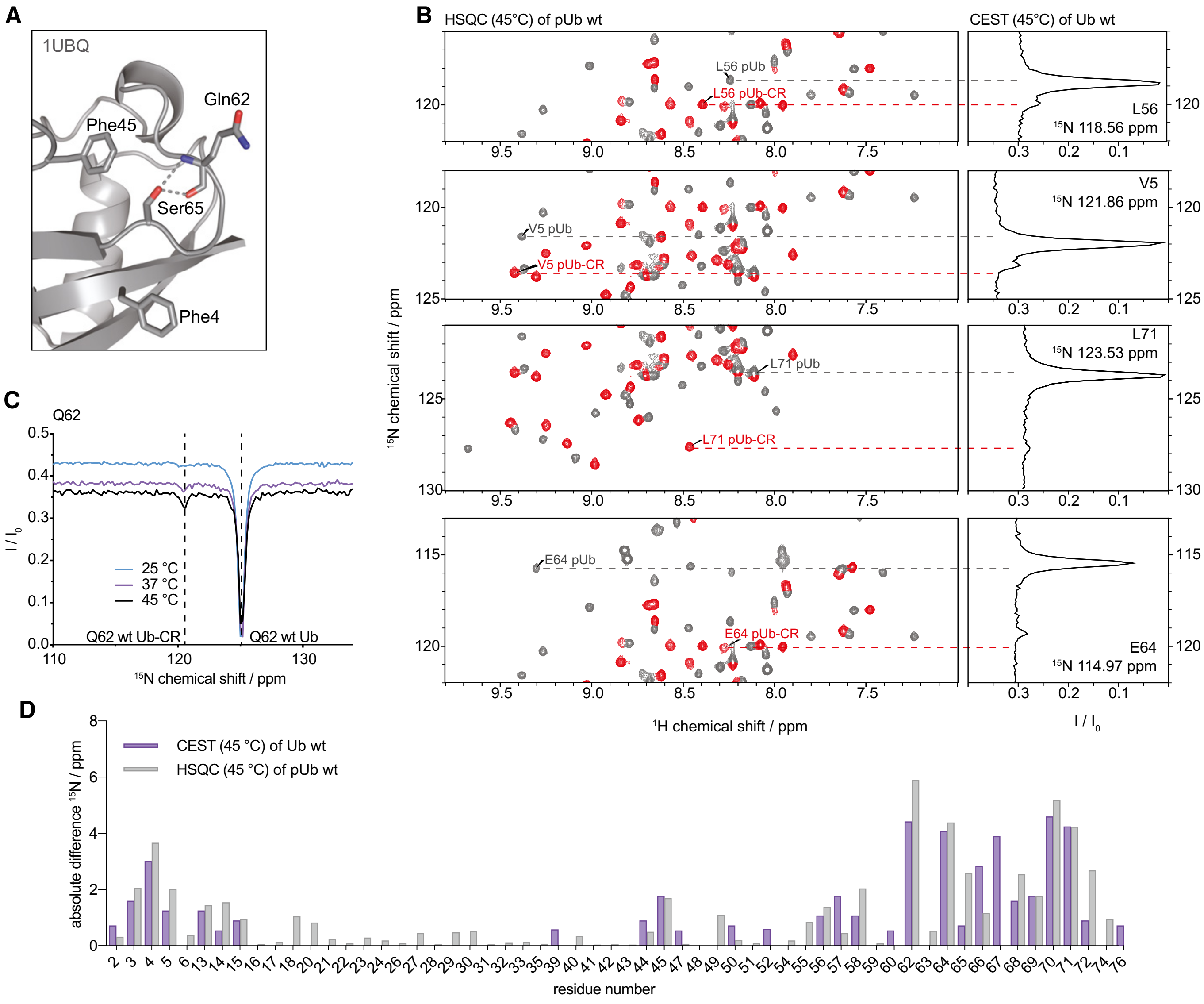

Figure EV1. CEST characterisation of wt Ub.

A Structure of the Ser65 loop in the common conformation of wt Ub (1UBQ; Vijay-Kumar *et al*, 1987), also showing nearby aromatic residues. The Ser65 hydrogen bonds are indicated.

B Additional CEST peaks as shown in Fig 1C. For full spectra, see Appendix Fig S1.

C Temperature dependence of CEST experiments for a selected resonance (Gln62). A Ub-CR CEST peak is clearly observed at 37°C but more pronounced at 45°C.

D Absolute ^{15}N-positional difference plot for all resonances according to Ub CEST at 45°C, in direct comparison with the ^{15}N-positional difference for each phosphoUb/phosphoUb-CR resonance pair in the phosphoUb HSQC spectrum at the same temperature.

© 2017 MRC Laboratory of Molecular Biology

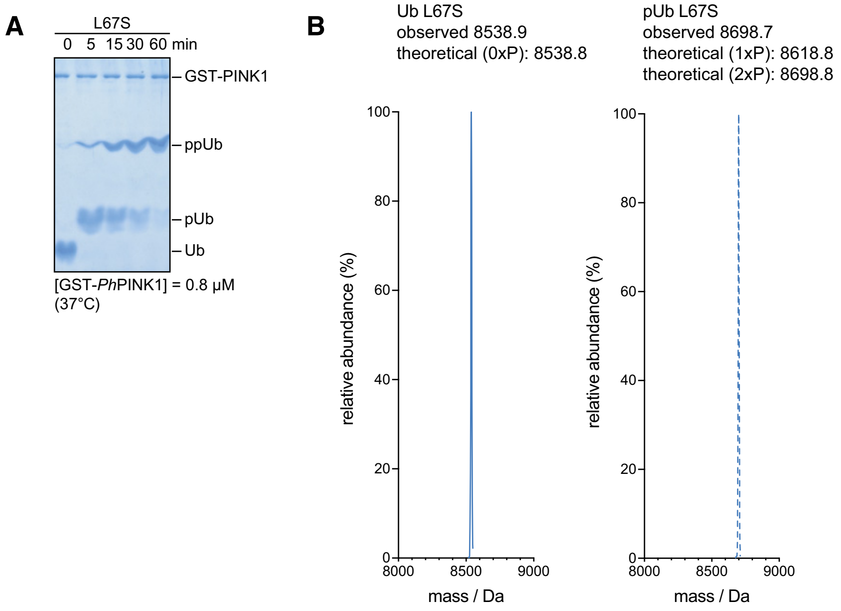

Figure EV2. Phosphorylation of Ub L67S.

A Phos-tag gel following Ub L67S phosphorylation by *Ph*PINK1. Ub L67S is phosphorylated twice. Data shown are representative of experiments performed in triplicate.

B LC-MS intact mass analysis of Ub L67S in unphosphorylated (left) and phosphorylated states (right). Two phosphorylation sites are observed.

 © 2017 MRC Laboratory of Molecular Biology

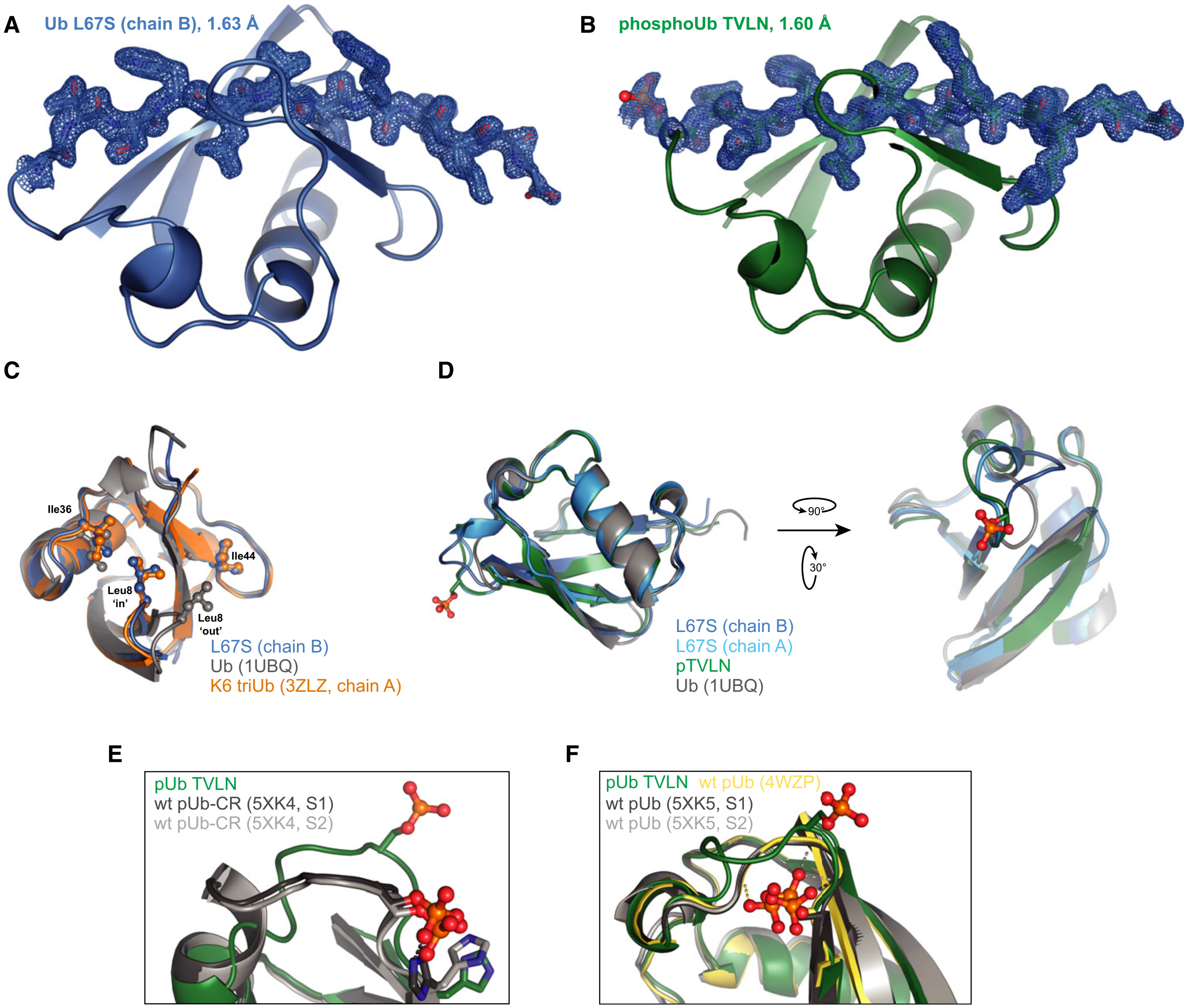

Figure EV3. Ub-CR crystal structures and comparison.

A Ub L67S in a view as in Fig 3A, showing $2|F_o|-|F_c|$ electron density at 1σ for the β5-strand.
B As in (A), for phosphoUb TVLN.
C Superposition of Ub with distinct positions of the β1–β2 loop. Unlike the common Ub conformation, where this loop is predominantly in the "out" position and Leu8 contributes to the Ile44 patch, the β1–β2 loop is in the "in" position in Ub-CR mutants such as Ub L67S; in this position, Leu8 contributes to the Ile36 patch. This loop conformation has also been observed in a subset of crystal structures, such as in Lys6-linked triUb (3ZLZ). See Hospenthal *et al* (2013) for further analysis.
D Two additional views of Ub-CR structures, highlighting the differences in the Ser65-containing loop.
E Comparison of phosphoUb TVLN with NMR structures of phosphoUb-CR (5XK4; Dong *et al*, 2017).
F Comparison of phosphoUb TVLN and phosphoUb crystal structures (4WZP; Wauer *et al*, 2015a) with NMR structures of phosphoUb (5XK5; Dong *et al*, 2017).

Figure EV4. Stability measurement of Ub-CR variants.

A Differential scanning calorimetry endotherms of indicated Ub variants in unphosphorylated and phosphorylated forms. A table summarising fitted melting parameters of each species is shown. Similar values for ΔH_{cal} and $\Delta H_{van't\ Hoff}$ indicate lack of intermediates during unfolding.
B $^{15}N\ \{^1H\}$ hetNOE experiment for Ub species in the common conformation (left) or Ub-CR (right) conformation, focussing at the flexible C-terminal tail. Large values are indicative of higher stability, and small or negative values indicate more flexibility. Values obtained from technical replicates are plotted individually. See Appendix Fig S6 for the complete datasets. Data for pUb and pUb-CR WT are replotted from Wauer *et al* (2015a).

© 2017 MRC Laboratory of Molecular Biology

A

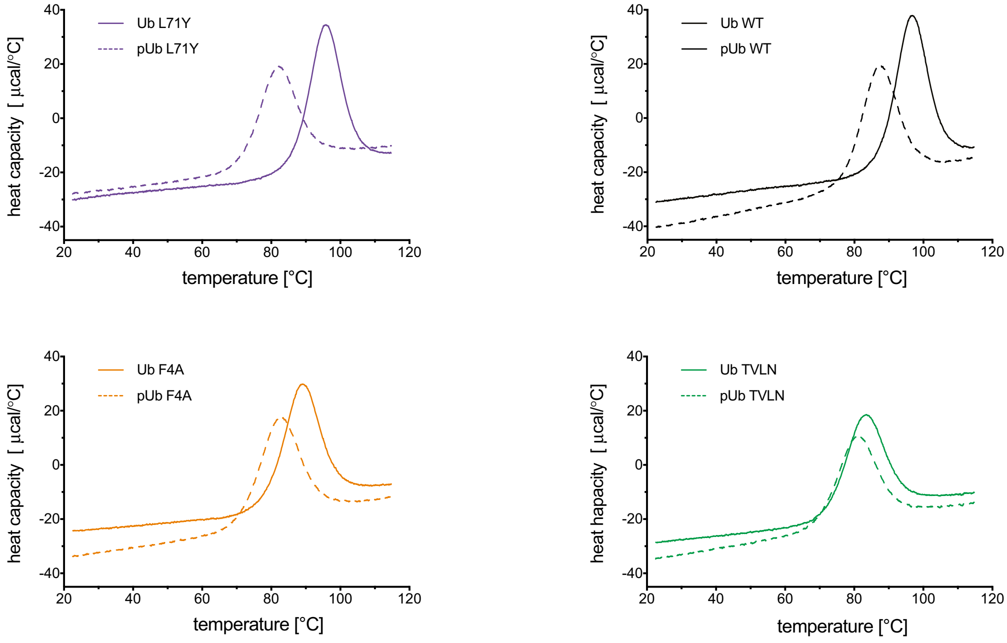

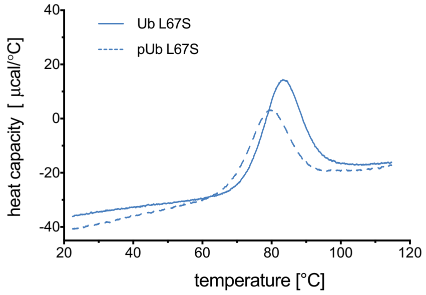

		T_m ± 0.2 °C	ΔH_{cal} (kcal/mol)	ΔH_{VH} (kcal/mol)
**wt**	Ub	96.5	94	91
	pUb	89.4	85	75
**L67S**	Ub	83.1	77	71
	pUb	79.6	60	66
**TVLN**	Ub	83.1	77	69
	pUb	80.9	67	68
**L71Y**	Ub	95.5	89	90
	pUb	81.8	72	71
**F4A**	Ub	88.7	84	77
	pUb	82.4	83	67

B

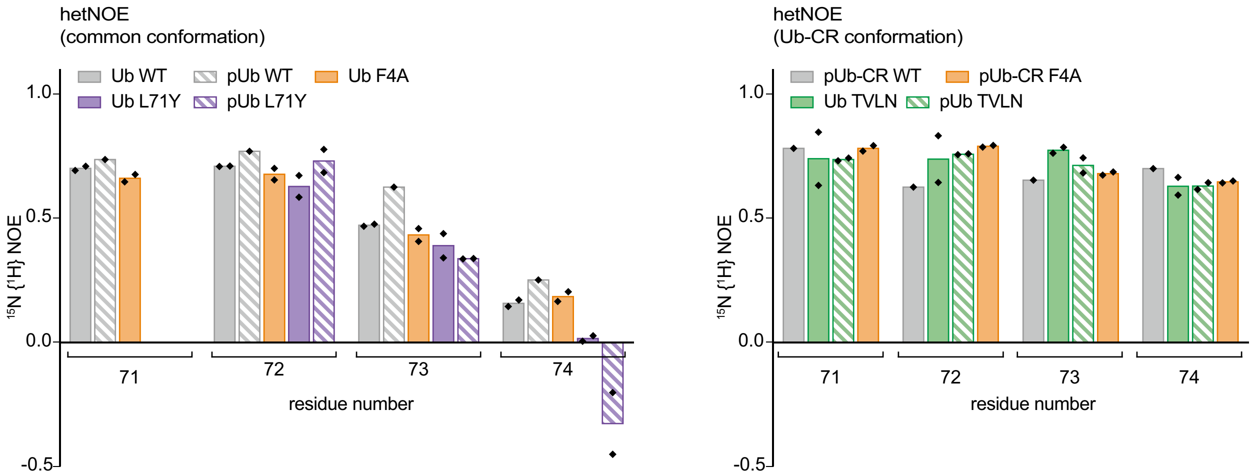

Figure EV4.

 © 2017 MRC Laboratory of Molecular Biology

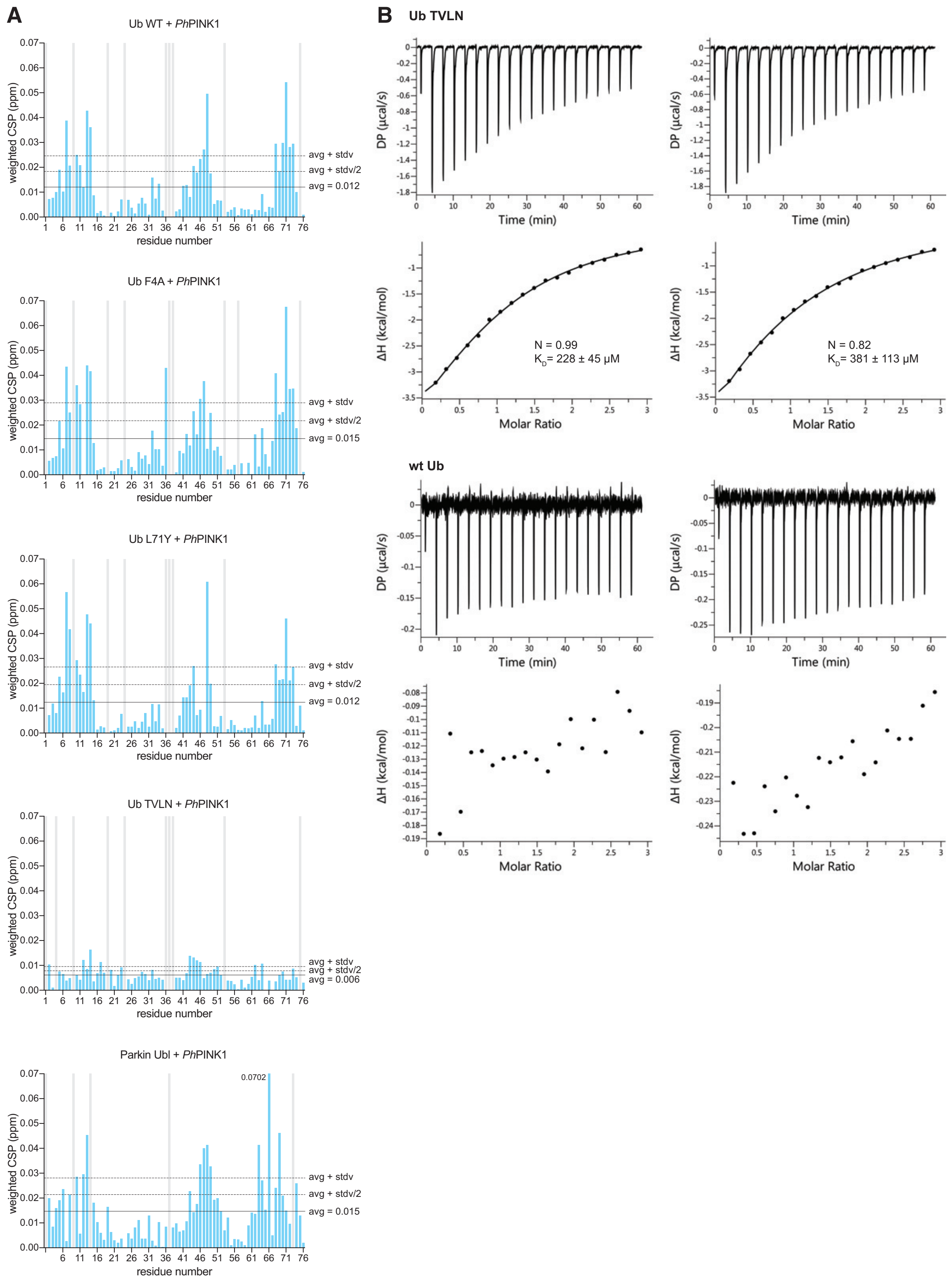

Figure EV5. PINK1 interaction with Ub variants.

A Weighted chemical shift perturbation analysis for ^{15}N-labelled wt Ub, Ub mutants and Parkin Ubl in the presence of *Ph*PINK1.

B Isothermal calorimetry titration analysis of wt or TVLN Ub into *Ph*PINK1. Data from two technical replicates are shown.

© 2017 MRC Laboratory of Molecular Biology

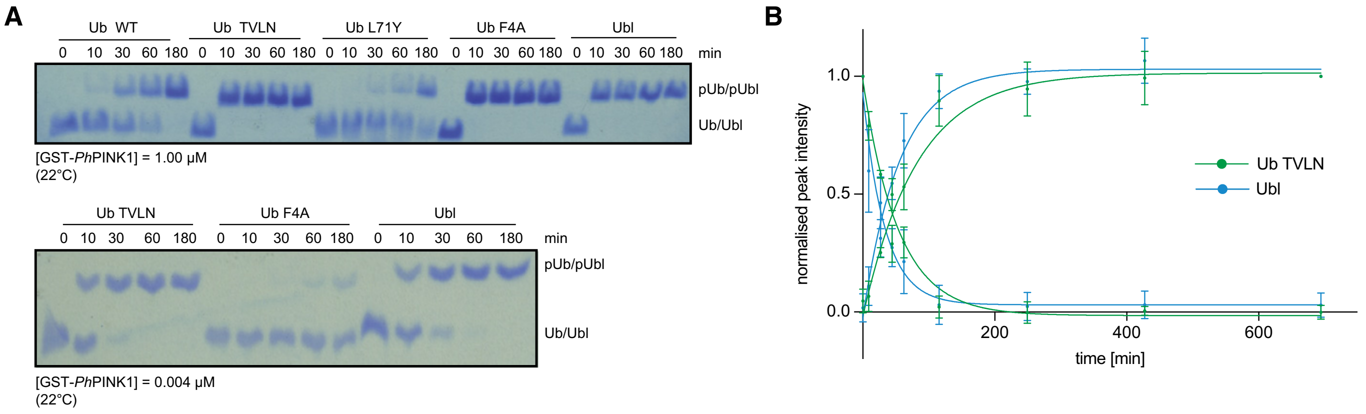

Figure EV6. Ub phosphorylation experiments by Phos-tag analysis.

A Analysis of substrate phosphorylation by *Ph*PINK1, using Phos-tag gels. A lowered concentration of *Ph*PINK1 is used to reveal similar kinetics of Ub TVLN and Parkin Ubl phosphorylation (bottom gel). Data shown are representative of experiments performed in triplicate.

B NMR-based Ub TVLN and Parkin Ubl phosphorylation as in Fig 8, but at lower *Ph*PINK1 enzyme concentration of 20 nM. Data from at least four individual resonances were averaged with error bars indicating standard deviation from the mean.

 © 2017 MRC Laboratory of Molecular Biology
